# Clinicians who think scapular dyskinesis is important are more likely to identify it in healthy individuals

**DOI:** 10.1177/17585732261424438

**Published:** 2026-02-23

**Authors:** Oscar Vila-Dieguez, Abel Cazorla-Rey, Lori A. Michener

**Affiliations:** 1 Department of Orthopaedic Surgery, 8784 University of California San Diego, La Jolla, California, USA; 2 Gimbernat Physical Therapy School, University of Cantabria, Torrelavega, Cantabria, Spain; 3 Division of Biokinesiology and Physical Therapy, 6889 University of Southern California, Los Angeles, California, USA

**Keywords:** Shoulder, scapular movement, interrater reliability, confirmation bias

## Abstract

**Objective:**

To assess interrater reliability of the visual scapular dyskinesis test among physical therapists and examine how clinician beliefs influence classifications of healthy shoulders.

**Methods:**

104 physical therapists (≥3 years of experience) rated five videos of healthy volunteers performing weighted arm elevations, using a Yes/No classification for scapular dyskinesis. Participants completed a survey rating the perceived importance of scapular dyskinesis (1–10 scale), reported years of experience, and number of shoulder patients seen weekly. Fleiss’ Kappa quantified reliability, and a mixed-effects model evaluated associations between ratings and clinician factors.

**Results:**

Interrater reliability was low (Fleiss’ Kappa = 0.12); agreement ranged from 53.9% to 76.9%. Clinicians who considered scapular dyskinesis more important were more likely to classify it as present (OR = 1.42; 95% CI [1.14, 1.76]; *p* = .0015), while those seeing more shoulder patients weekly were less likely to do so (OR = 0.77; 95% CI [0.62, 0.94]; *p* = .021). Experience did not predict classification.

**Conclusion:**

The visual scapular dyskinesis test demonstrated poor reliability. Beliefs about its clinical importance and lower shoulder caseloads increased the likelihood of reporting dyskinesis. These findings question the test's diagnostic value and underscore the need for more objective assessment methods.

## Introduction

Scapular dyskinesis is defined as an alteration in the normal position or motion of the scapula during coupled scapulohumeral movements,^1^ and has been widely investigated in shoulder pathologies.^2^ Research interest in scapular dyskinesis has grown substantially, with PubMed indexing 120 publications between 2005 and 2015 and 327 between 2015 and 2025.^3^ While scapular dyskinesis is often cited as a contributing factor in shoulder pain,^4–6^ especially in athletic populations,^7,8^ its presence is also common in healthy individuals,^2,8^ raising questions about its clinical relevance.

Among the many clinical tools developed to identify scapular dyskinesis, observational tests remain the most practical in clinical settings,^9^ but the reliability and accuracy of visual assessment are still a concern.^10^ The scapular dyskinesis test, as proposed by Uhl^11^ involves visual inspection of scapular movement during shoulder flexion or abduction and classifying the movement as either normal or dyskinetic (“Yes/No” method). This test is widely used due to its relative simplicity and has shown moderate interrater agreement (*κ* = 0.40; agreement = 79%) and sensitivity (76%).^11^ A three-level rating (none, subtle, and obvious dyskinesis) has also demonstrated moderate reliability between clinician raters (*κ* = 0.48–0.61; agreement = 75%–82%)^12^ and concurrent validity compared to three-dimensional motion capture.^13^

Two systematic reviews investigating the reliability of visual scapular dyskinesis assessment concluded that there is a lack of high-quality studies and more research is required to recommend its clinical use.^14,15^ The main limitation in current reliability studies is that they involve small samples of raters, typically 2–3 clinicians from the same institution.^14,15^ This limits generalizability and describes the reliability for raters that may share similar beliefs regarding the importance of scapular dyskinesis. Plummer et al.^16^ showed that when raters knew a participant had shoulder symptoms, they were more likely to report the presence of scapular dyskinesis, suggesting that observational tests can be affected by clinician confirmation bias. However, even without knowing whether a patient has symptoms, a clinician's preexisting beliefs about the importance of scapular movement may bias their assessment. English philosopher Francis Bacon^17^ described the phenomenon that was later called “confirmation bias” as follows: “The human understanding when it has once adopted an opinion draws all things else to support and agree with it, in order that by this great and pernicious predetermination the authority of its former conclusions may remain inviolate.”

The present study aims to evaluate the interrater reliability of the visual scapular dyskinesis test when used by a large group of physical therapists assessing standardized video cases. By inverting the typical design—using few patients and many raters—we aim to assess the generalizability of test reliability across clinicians. Furthermore, we explore how clinicians’ beliefs about the importance of scapular dyskinesis and their experience with managing patients with shoulder pain influences classification decisions. We hypothesized that interrater reliability would be lower than in previous studies and that raters who consider scapular dyskinesis to be more important would report the presence of scapular dyskinesis more frequently.

## Methods

### Study Design

This observational cross-sectional study was conducted and reported in accordance with the Strengthening the Reporting of Observational Studies in Epidemiology guidelines.

### Participants

Physical therapists (*n* = 104) from various countries through social media platforms engaged and participated in this study. Participant inclusion criteria were: (1) licensed physical therapists in their country of practice, (2) minimum of three years of clinical experience, (3) treat a minimum of five patients per week with shoulder pathology. Prior to participation, participants provided informed consent. This study was approved by the Institutional Review Board of the University of Cantabria.

### Procedure

The data collection was performed online using Google Forms and was organized into three sections. The first section gathered demographic and professional background information to verify eligibility. Participants reported their country of current practice, years of experience, clinical specialization, and estimated number of weekly patients seen with shoulder pathology. These were included as potential covariates that could influence scapular dyskinesis classification.

Section two assessed participants’ beliefs and perceived importance of scapular dyskinesis in their clinical practice through a custom-developed survey for this study. Respondents rated their agreement on a Likert scale from 1 to 10 (1 being “*strongly disagree*” and 10 being “*strongly agree*”) with the following statements:

1. “Scapular kinematics should always be observed in every patient with shoulder symptoms.”
2. “Based on my clinical experience, most of the scapular movement asymmetries I observe are relevant to the patient's condition.”
3. “If I identify scapular dyskinesis in a patient, part of my treatment will focus on improving scapular stability.”
4. “In general, I pay close attention to scapular movement regardless of the type of shoulder injury.”

Then for section three, participants completed an observational analysis of five video recordings of healthy volunteers. Video recordings were conducted as previously described that have shown reliability comparable to live assessment.^12,18^ The camera was positioned on a tripod, at a distance of 1 to 1.5 m from the participant, adjusted based on the participant's height to ensure clear visualization of scapular motion. The center of the lens was placed approximately at the T3 level of the thoracic spine. Volunteers were barefoot, and shirtless or wearing a sports bra. Each volunteer performed five repetitions of shoulder abduction followed by five repetitions of shoulder flexion. Prior to filming, each volunteer practiced five repetitions of each movement to ensure consistency. Movements were performed through a full range of shoulder motion at a controlled tempo, with both concentric and eccentric phases lasting 5 s. Each volunteer held a 1.4 or 2.3 kg weight in each hand (under or over 68.1 kg body weight respectively), consistent with previous literature that used weight to exacerbate potential movement deviations.^12,18^

To avoid confounding factors such as knowledge of patient symptoms, clinicians were blinded to symptom status (all participants in the videos were asymptomatic). Each clinician reviewed videos and classified whether the individual presented with scapular dyskinesis (“Yes”) or not (“No”) following the classification and guidelines described by Uhl^11^: “Yes” included one or more of these features: prominence of the inferior medial scapular angle associated with excessive anterior tilting of the scapula, prominence of the entire medial border associated with excessive scapular internal rotation and/or prominence of the superior scapular border associated with excessive upward translation of the scapula. “No” indicated that no asymmetries were identified, and no prominence of the medial or superior border was observed. Normal scapular motion was described as bilateral posterior tilting, external rotation, and slight superior translation during arm elevation and reversal of these during lowering relative to the opposite side.

### Statistical analysis

Clinician agreement in classifying Yes/No to the presence of scapular dyskinesis was assessed using Fleiss’ Kappa, a chance-corrected measure of interrater reliability suitable for multiple raters assigning categorical ratings to multiple items.^19^ Kappa values range from −1 (*complete disagreement*) to 1 (*perfect agreement*), with values closer to 0 indicating agreement no better than chance. In addition to kappa, the percent agreement was calculated for each video. This was defined as the proportion of clinicians who selected the majority response (Yes/No), providing a measure of agreement without adjusting for chance. Ninety-five percent confidence intervals (95% CIs) for percent agreement were computed as binomial proportion confidence intervals using the Wilson score method.^20^ The number of clinicians endorsing each response category was also summarized per video.

To evaluate the association between the classification of scapular dyskinesis and three rater-level covariates (years of practice, volume of shoulder patients seen, belief of the importance of scapular dyskinesis), a generalized linear mixed-effects model with a binomial distribution and logit link function was used.^21^ The dependent variable was the presence or absence of scapular dyskinesis (Yes/No). Fixed effects included years of practice, volume of shoulder patients seen weekly, and the importance attributed to scapular dyskinesis (all z-scored to adjust for different magnitudes). Clinician rater was included as a random intercept to account for clustering of responses within raters. The model was fit using maximum likelihood estimation via Laplace approximation.^22^ Statistical significance was defined as *p* < .05. All analyses were performed in R using the lme4 package. Model assumptions were assessed using the DHARMa package, which simulates standardized residuals for generalized linear mixed-effects models.^23^

## Results

**Figure 1.**
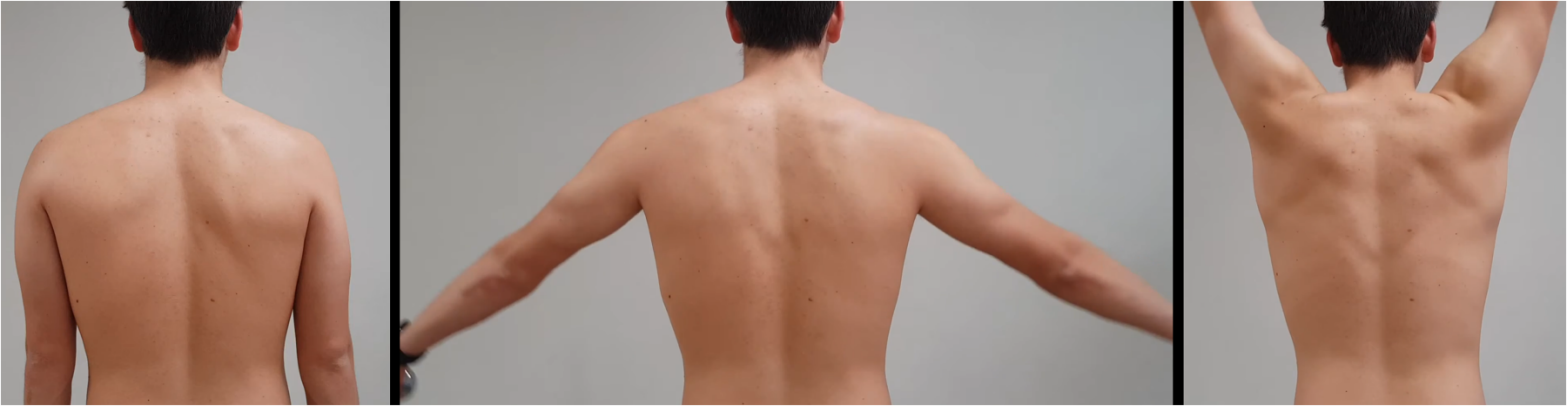
Appearance of the videos in the questionnaire.

One hundred four physical therapists participated in this study. They reported having 6.6 ± 3.5 years of clinical experience and seeing 10 ± 5.2 patients with shoulder complaints per week. The average score for the scapular dyskinesis importance survey was 21.7 ± 10 out of 40 points. Table 1 includes all other characteristics of the participants. Across the five videos, 50% of the ratings were for positive dyskinesis and 50% for negative. Interrater agreement was low, with a Fleiss’ Kappa of 0.12, indicating slight agreement beyond chance. When examined individually, percent agreement varied across videos. Percent agreement ranged from 53.9% to 76.9%, with the highest agreement observed for Video 1 and Video 5. Table 2 summarizes the number of clinicians rating each video as normal or abnormal, along with the calculated percent agreement (Figure 1).

**Table 1.** Characteristics of clinicians.

		*N*	%
Country	Spain	46	44.2
	United Kingdom	13	12.5
	United States	10	9.6
	Netherlands	8	7.7
	Argentina	4	3.8
	France	4	3.8
	Australia	3	2.9
	Canada	3	2.9
	Belgium	2	1.9
	Switzerland	2	1.9
	Sweden	1	1
	Germany	1	1
	Ireland	1	1
	India	1	1
	Slovakia	1	1
	Norway	1	1
	South Africa	1	1
	Paraguay	1	1
	Turkey	1	1
Patient population*	Orthopedics/ neuromusculoskeletal	91	87.5
	Sport	31	29.8
	Geriatrics	7	6.7
	Neurology	3	2.9
	Other	3	2.9
	Oncology	2	1.9

*More than one answer possible.

**Table 2.** Yes/no classification count per video and interrater agreement.

Video	No (0)	Yes (1)	Percent agreement
1	80	24	76.9% (68.0%, 84.0%)
2	61	43	58.7% (49.0%, 67.6%)
3	34	70	67.3% (57.8%, 75.6%)
4	56	48	53.9% (44.3%, 63.1%)
5	29	75	72.1% (62.8%, 79.8%)
Average	52	52	65.8% (61.6%, 69.7%)

**Figure 2.**
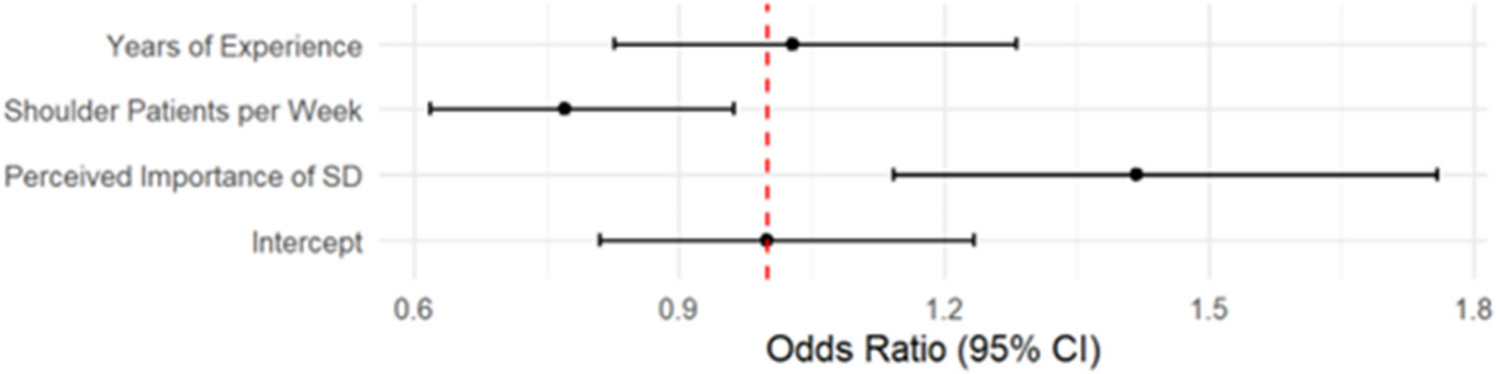
Forest plot of the odds ratios and 95% confidence intervals for all fixed effects.

**Figure 3.**
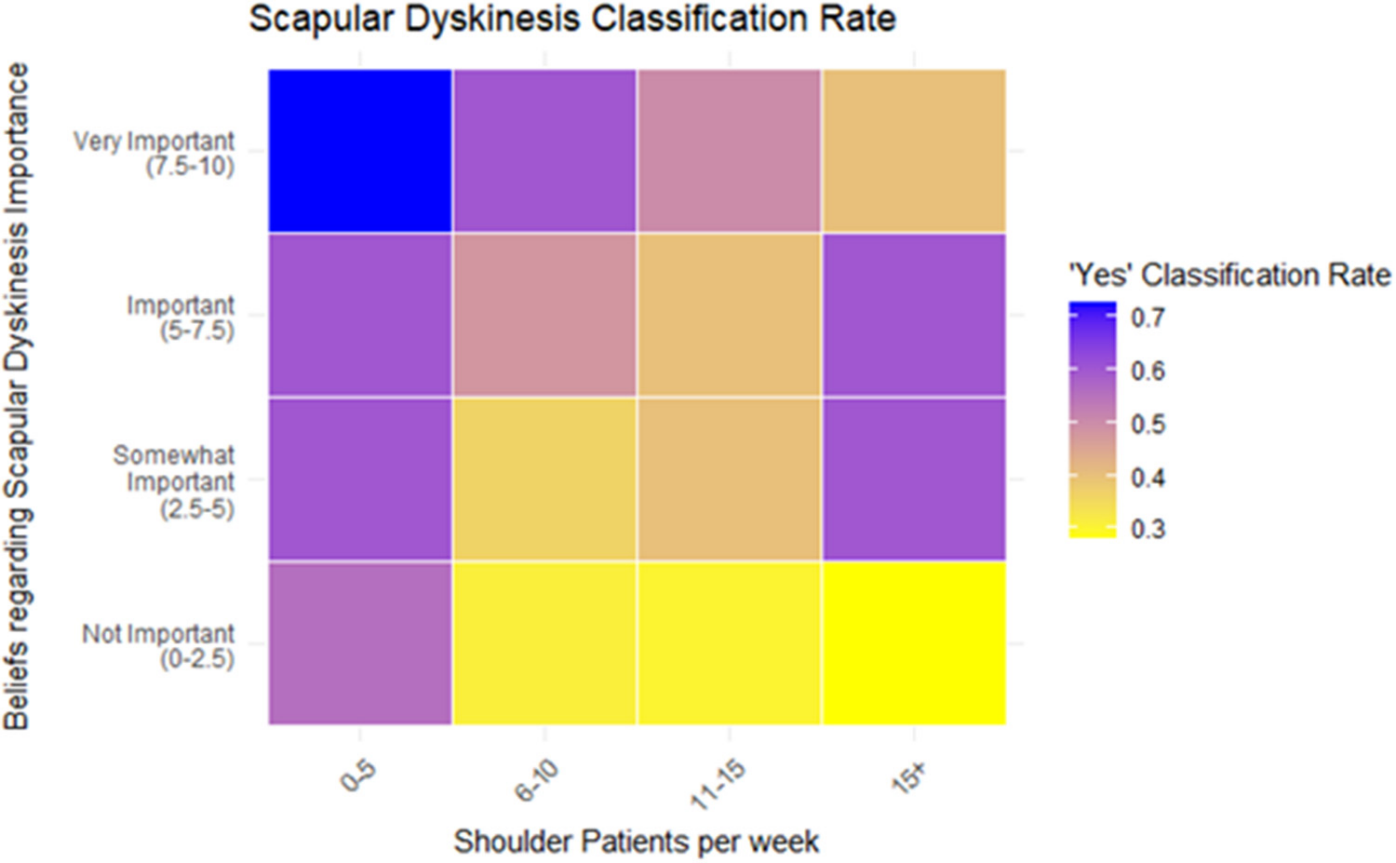
Heatmap representing the impact of beliefs regarding scapular dyskinesis importance and shoulder patients seen per week in the rate of positive scapular dyskinesis reported. Those who rated highly their perceived importance of scapular dyskinesis and see less patients with shoulder pathology were more likely to report the presence of scapular dyskinesis.

The mixed effects model showed that perceived importance of scapular dyskinesis was significantly associated with the likelihood of reporting the presence of scapular dyskinesis (β = 0.35, SE = 0.11, *z* = 3.17, *p* = .0015), corresponding to an odds ratio of 1.42 (95% CI [1.14, 1.76]) (Figure 2). Conversely, the number of shoulder patients seen weekly was negatively associated with reporting the presence of scapular dyskinesis (β = –0.26, SE = 0.11, *z* = –2.31, *p* = .021), with an odds ratio of 0.77 (95% CI [0.62, 0.94]). Years of experience treating shoulder conditions was not a significant predictor (β = 0.03, *p* = .80), with an odds ratio of 1.03 (95% CI [0.83, 1.28]). Random effect variance for clinician rater was 0.303, indicating modest variability in classification tendencies across raters. Residual diagnostics using DHARMa indicated no significant violations of model assumptions (KS test *p* = .062; dispersion test *p* = .848; outlier test *p* = .318), supporting the adequacy of model fit. Figure 3 displays a heatmap illustrating the two significant fixed effects from the mixed-effects model: perceived importance of scapular dyskinesis (*y*-axis) and number of shoulder patients seen per week (*x*-axis), each divided into four categories. The color gradient represents the average rate of “Yes” classification for scapular dyskinesis within each category.

SD: Scapular dyskinesis.

## Discussion

This study evaluated the interrater reliability of the Yes/No scapular dyskinesis test using a large international sample of physical therapists assessing standardized video recordings. In contrast to earlier studies that reported moderate inter- and intratester agreement with this test among small groups of clinicians from similar settings,^14,15^ our findings showed only slight agreement beyond chance. However, prior studies that included more than three clinician raters had lower reliability metrics than studies with two or three raters^12,24,25^ Ellenbecker et al.^24^ found Kappas ranging from 0.08 to 0.26 with four raters, comparable to our findings. Kibler et al.^25^ found Kappas ranging from 0.31 to 0.42, slightly superior to our findings, but lower than studies with two and three raters. Finally, McClure et al.^12^ reported kappas ranging from 0.29 to 0.78 with six raters (three pairs). These results are higher than in the present study but also lower compared to studies with two and three raters. This highlights the potential decrease in reliability as more raters are added with different clinical backgrounds and different beliefs regarding the importance of scapular dyskinesis, as evidenced by the present study.

Percent agreement ranged from 53.9% to 76.9% across videos. The inconsistency across raters reinforces prior conclusions from systematic reviews that questioned the clinical utility of visual scapular tests.^2,10^ It is critical to emphasize that, even though the videos were recorded with healthy volunteers in this study, 50% of the responses indicated that scapular dyskinesis was present. This finding is consistent with prior literature that finds a 42% to 59% of cases are reported as scapular dyskinesis among asymptomatic individuals.^2^

A key contribution of this study is the finding that clinicians’ preconceived beliefs about scapular dyskinesis influenced the rating of the presence of scapular dyskinesis. The likelihood of assigning a Yes classification was positively associated with the perceived importance of scapular dyskinesis. These findings point to the influence of confirmation bias—clinicians who believe scapular dyskinesis is important may be more prone to seeing it. This aligns with the findings by Plummer,^16^ who demonstrated that clinicians were more likely to report the presence of scapular dyskinesis when they knew the participant had shoulder pain versus when they did not know if shoulder pain was present.

Clinicians who see more shoulder patients per week were less likely to indicate the presence of dyskinesis. It is possible that these clinicians may apply different criteria for the presence of scapular dyskinesis or may have become desensitized to small variations in scapular motion. These are commonly seen across patients with shoulder pain and do not necessarily change after intervention despite improvements in shoulder pain and disability.^2,26^ In addition, the current criteria for the Yes/No scapular dyskinesis classification is highly subjective, as there are no indications of what a normal range is for the features that are presented as “dyskinetic.” There are no objective terms for how much the inferior, medial or superior borders of the scapula have to protrude for it to be considered a dyskinetic pattern. Even if there were such objective indications, they would not be measurable through visual assessment. Interestingly, years of experience did not predict classification, suggesting that frequency of exposure to shoulder pathology, rather than time in practice, may be a more influential factor in shaping clinical decision-making.

Together, these findings raise critical questions about the role of the visual scapular dyskinesis test, although this study can only support this claim in the context of healthy individuals. While it remains a practical and low-cost tool, its low reliability and susceptibility to bias limits the value as a standalone test. Visual assessment should instead be interpreted within a broader clinical context that includes identifying shoulder movements or tasks that are specific to the patient as opposed to generic movement quality assessment approaches, along with symptom provocation to determine if alteration in scapular motion changes patient symptoms. Also, other objective measures may be helpful, such as psychological factors, systemic disease and pain processing.

### Limitations

Several limitations should be acknowledged. First, although the use of standardized video recordings improves consistency of scapular motion assessed across raters and has been validated previously,^12,18^ it cannot fully replicate the nuances of live clinical examination, including palpation and dynamic interaction. Second, recruitment via social media may introduce self-selection bias and may not represent all clinical backgrounds or regions. Almost half of the sample practiced in Spain, which may not share the same training, culture or healthcare system with other countries, limiting the applicability of these findings to all countries. Finally, with only five cases assessed, and all of them being healthy individuals, the range of scapular movement patterns may not capture the full spectrum of the clinical presentation of scapular dyskinesis. It is possible that in the presence of pathologies that impact scapular motion significantly, such as long thoracic nerve palsy, reliability across a large number of raters is better compared to this study and beliefs regarding scapular dyskinesis do not affect rating to the same extent.

## Conclusion

The Yes/No scapular dyskinesis test demonstrated poor interrater reliability when applied across a diverse international sample of physical therapists. Classification decisions were influenced by clinicians’ beliefs regarding the importance of scapular dyskinesis and the volume of exposure to shoulder patients, suggesting that confirmation bias and experience play a role in visual interpretation of scapular dyskinesis. These findings call into question the clinical utility of the scapular dyskinesis test as a standalone tool to identify scapular deficits and support the need for other assessment strategies to determine the relevance of deficits in visual scapular movement.

## Key points

### Findings

The visual scapular dyskinesis test demonstrated poor interrater reliability when applied across 104 physical therapists from different countries and clinical settings. Clinicians who considered scapular dyskinesis to be more important in their practice were more likely to report its presence in healthy individuals. In addition, those who treated less shoulder patients per week were also more likely to report the presence of scapular dyskinesis.

### Implications

The scapular dyskinesis test appears to be influenced by clinicians’ beliefs and clinical exposure to patients with shoulder pathology, raising concerns about confirmation bias. Given the limited agreement among raters and influence of clinician factors, the test should not be used in isolation to guide clinical decision-making.

### Caution

Although standardized video recordings were used to ensure consistency, they may not fully capture the nuances of live assessment. The inclusion of only five healthy cases may limit generalizability across the full spectrum of clinical presentations.

## Supplemental Material

sj-docx-1-sel-10.1177_17585732261424438 - Supplemental material for Clinicians who think scapular dyskinesis is important are more likely to identify it in healthy individuals Supplemental material, sj-docx-1-sel-10.1177_17585732261424438 for Clinicians who think scapular dyskinesis is important are more likely to identify it in healthy individuals by Oscar Vila-Dieguez, Abel Cazorla-Rey and Lori A. Michener in Shoulder & Elbow
