# Supplementary material for: Clinicians who think scapular dyskinesis is important are more likely to identify it in healthy individuals
Source: Shoulder Elbow. 2026 Feb 23:17585732261424438. Online ahead of print. doi: 10.1177/17585732261424438 (PMC12929090; doi:10.1177/17585732261424438)
Supplement: sj-docx-1-sel-10.1177_17585732261424438 - Supplemental material for Clinicians who think scapular dyskinesis is important are more likely to identify it in healthy individuals [file sj-docx-1-sel-10.1177_17585732261424438.docx]

**Supplementary Material 1**

**SCAPULAR DYSKINESIS QUESTIONNAIRE**

**By clicking on “YES” you certify to be a fully licensed Physical Therapist, and you agree to the future use of the data in this study (for research purposes), which is completely anonymous:**

Yes

**----------------------------------------------------------------------------------------------------------------**

**Current country of practice:**

**How many years have you been working as a physical therapist?**

**In which field of physical therapy are you specialized or currently working at?**

Orthopedics/Neuromusculoskeletal

Neurology

Geriatrics

Pediatrics

Sport

Oncology

Other

**Approximately, how many patients with shoulder symptoms do you see per week?**

----------------------------------------------------------------------------------------------------------------

**Rate from 1 (low) to 10 (high) your agreement with the following four statements:**

**1.-Scapular kinematics should always be observed in every patient with shoulder symptoms.**

1 2 3 4 5 6 7 8 9 10

**2.- Based on my clinical experience, most of the scapular movement asymmetries I observe are relevant to the patient’s condition.**

1 2 3 4 5 6 7 8 9 10

**3.- If I identify scapular dyskinesis in a patient, part of my treatment will focus on improving scapular stability.**

1 2 3 4 5 6 7 8 9 10

**4.- In general, I pay close attention to scapular movement regardless of the type of shoulder injury.**

1 2 3 4 5 6 7 8 9 10

**----------------------------------------------------------------------------------------------------------------**

**Watch these videos and classify each according to the criteria by Uhl and colleagues:** “Yes” included one or more of these features: prominence of the inferior medial scapular angle associated with excessive anterior tilting of the scapula, prominence of the entire medial border associated with excessive scapular internal rotation and/or prominence of the superior scapular border associated with excessive upward translation of the scapula. “No” indicated that no asymmetries were identified and no prominence of the medial or superior border was observed. Normal scapular motion was described as bilateral posterior tilting, external rotation, and slight superior translation during arm elevation and reversal of these during lowering relative to the opposite side

Choose YES if the person shows scapular dyskinesis or NO if they do not.
Watch each video more than once if necessary.
